# Crystal structure of a sodium, zinc and iron(III)-based non-stoichiometric phosphate with an alluaudite-like structure: Na_1.67_Zn_1.67_Fe_1.33_(PO_4_)_3_

**DOI:** 10.1107/S2056989015009767

**Published:** 2015-05-23

**Authors:** Jamal Khmiyas, Abderrazzak Assani, Mohamed Saadi, Lahcen El Ammari

**Affiliations:** a Laboratoire de Chimie du Solide Appliquée, Faculté des Sciences, Université Mohammed V, Avenue Ibn Battouta, BP 1014, Rabat, Morocco

**Keywords:** crystal structure, transition-metal phosphates, solid-state reaction synthesis, alluaudite structure type, Na_1.67_Zn_1.67_Fe_1.33_(PO_4_)_3_

## Abstract

The new title compound, disodium dizinc iron(III) tris­(phosphate), Na_1.67_Zn_1.67_Fe_1.33_(PO_4_)_3_, which belongs to the alluaudite family, has been synthesized by solid-state reactions. In this structure, all atoms are in general positions except for four, which are located on special positions of the *C*2/*c* space group. This structure is characterized by cation substitutional disorder at two sites, one situated on the special position 4*e* (2) and the other on the general position 8*f*. The 4*e* site is partially occupied by Na^+^ [0.332 (3)], whereas the 8*f* site is entirely filled by a mixture of Fe and Zn. The full-occupancy sodium and zinc atoms are located at the Wyckoff positions on the inversion center 4*a* (-1) and on the twofold rotation axis 4*e*, respectively. Refinement of the occupancy ratios, bond-valence analysis and the electrical neutrality requirement of the structure lead to the given composition for the title compound. The three-dimensional framework of this structure consists of kinked chains of edge-sharing octa­hedra stacked parallel to [10-1]. The chains are formed by a succession of trimers based on [ZnO_6_] octa­hedra and the mixed-cation Fe^III^/Zn^II^ [(Fe/Zn)O_6_] octa­hedra [Fe^III^:Zn^III^ ratio 0.668 (3)/0.332 (3)]. Continuous chains are held together by PO_4_ phosphate groups, forming polyhedral sheets perpendicular to [010]. The stacked sheets delimit two types of tunnels parallel to the *c* axis in which the sodium cations are located. Each Na^+^ cation is coordinated by eight O atoms. The disorder of Na in the tunnel might presage ionic mobility for this material.

## Abstract 2

The transition-metal orthophosphate Na_1.67_Zn_1.67_Fe_1.33_(PO_4_)_3_ cristallizes in an alluaudite-type structure. The chains characterizing the alluaudite structure are then built up from [*M*_2_O10] (*M* = Fe/Zn) units alternating with [ZnO_6_] octa­hedra. This structure is characterized by a cationic disorder in one tunnel and in the general position.

## Chemical context   

Alkali transition-metal phosphates belonging to the alluaudite family constitute one of the most diverse and rich classes of minerals, and have been studied intensively over the last few years. Owing to their outstanding physico-chemical properties, these compounds have many potential applications in various fields, such as catalytic activity (Kacimi *et al.*, 2005 ▸) and as promising cathodes for sodium-ion batteries through the presence of mobile cations located in the tunnels of the open three-dimensional framework (Huang *et al.*, 2015 ▸). In their recent study, Huang *et al.* (2015 ▸) point out that the electrochemical performance is not only associated with morphology, but also with the electronic and crystalline structure.

Accordingly, a large number of alluaudite phases with alkali cations in the tunnels have been reported. Nevertheless, the presence of alkali metals in the tunnels of synthetic alluaudite phases is frequently accompanied by cationic distributions that lead to non-stoichiometric compositions, such as: (Na_0.38_,Ca_0.31_)MgFe_2_(PO_4_)_3_ (Zid *et al.*, 2005 ▸); NaFe_3.67_(PO_4_)_3_ (Korzenski *et al.*, 1998 ▸); Cu_1.35_Fe_3_(PO_4_)_3_ (Warner *et al.*, 1993 ▸); K_0.53_Mn_2.37_Fe_1.24_(PO_4_)_3_ (Hidouri & Ben Amara, 2011 ▸); Na_1.79_Mg_1.79_Fe_1.21_(PO_4_)_3_ (Hidouri *et al.*, 2003 ▸); Na_1.50_Mn_2.48_Al_0.85_(PO_4_)_3_ (Hatert, 2006 ▸); Na_1−*x*_Li_*x*_MnFe_2_(PO_4_)_3_ where *x* = 0, 0.25, 0.50, and 0.75 (Hermann *et al.*, 2002 ▸). As part of our study on alluaudite-related phosphates (Bouraima *et al.*, 2015 ▸; Assani *et al.*, 2011 ▸), we report the synthesis and the crystal structure of a new sodium, zinc and iron-based non-stoichiometric phosphate, namely Na_1.67_Zn_1.67_Fe_1.33_(PO_4_)_3_.

## Structural commentary   

**Figure 1:**
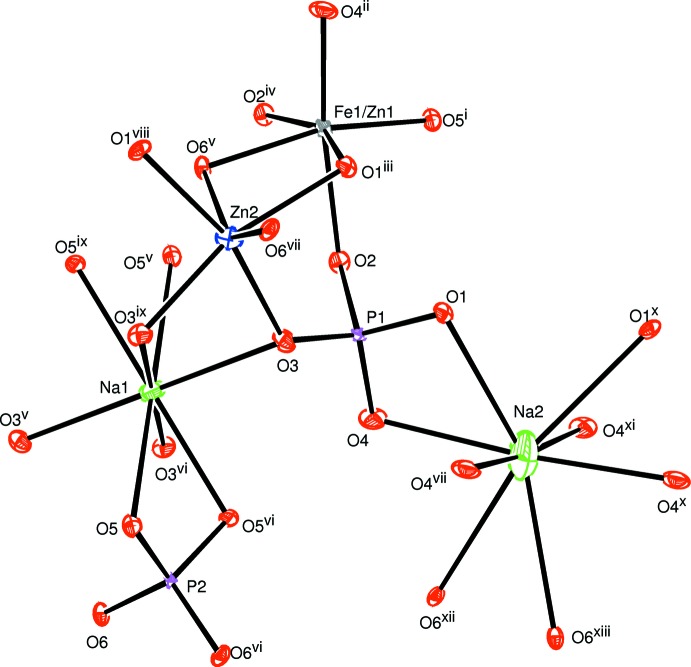
The principal building units in the structure of the title compound. Displacement ellipsoids are drawn at the 50% probability level. [Symmetry codes: (i) *x* + 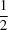, *y* + 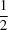, *z*; (ii) −*x* + 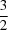, *y* + 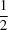, −*z* + 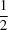; (iii) −*x* + 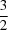, −*y* + 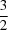, −*z* + 1; (iv) −*x* + 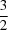, −*y* + 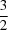, −*z*; (v) −*x* + 1, −*y* + 1, −*z*; (vi) −*x* + 1, *y*, −*z* + 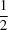; (vii) *x*, −*y* + 1, *z* + 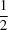; (viii) *x* − 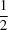, −*y* + 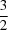, *z* − 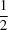; (ix) −*x* + 2, *y*, −*z* + 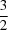; (*x*) −*x* + 2, −*y* + 1, −*z* + 1; (xi) *x* + 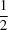, −*y* + 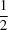, *z* + 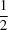; (xii) −*x* + 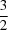, −*y* + 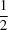, −*z* + 1; (xiii) *x*, −*y* + 1, *z* − 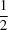.]

The alluaudite structure of the title compound crystallizes in the monoclinic space group *C*2/*c*, with Z = 4. The principal building units of the crystal structure are represented in Fig. 1 ▸. Refinement of the occupancy fractions, bond-valence analysis based on the formula proposed by Brown & Altermatt (1985 ▸) and the required electrical neutrality of the structure lead to the formula Na_1.67_Zn_1.67_Fe_1.33_(PO_4_)_3_ for the title compound. The mixed Fe1 and Zn1 atoms are located at the general position 8*f* with Fe^3+^/Zn^2+^ occupancy fractions of 0.668 (3)/0.332 (3), and form a highly distorted [(Fe1/Zn1)O_6_] octa­hedral group, with Fe^3+^/Zn^2+^—O bond lengths ranging from 1.951 (1) to 2.209 (1)Å. The Zn2 atom is surrounded by six oxygen atoms, building a slightly distorted octa­hedron with an average Zn2—O bond length of 2.153 (1) Å.

**Figure 2:**
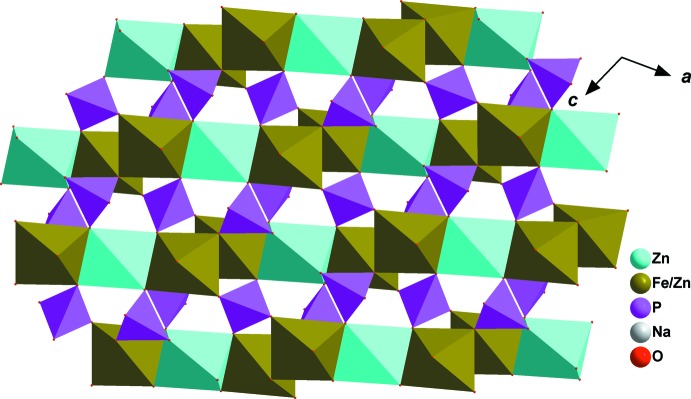
A view along the *b* axis of a sheet resulting from chains connected by vertices of PO_4_ tetra­hedra.

**Figure 3:**
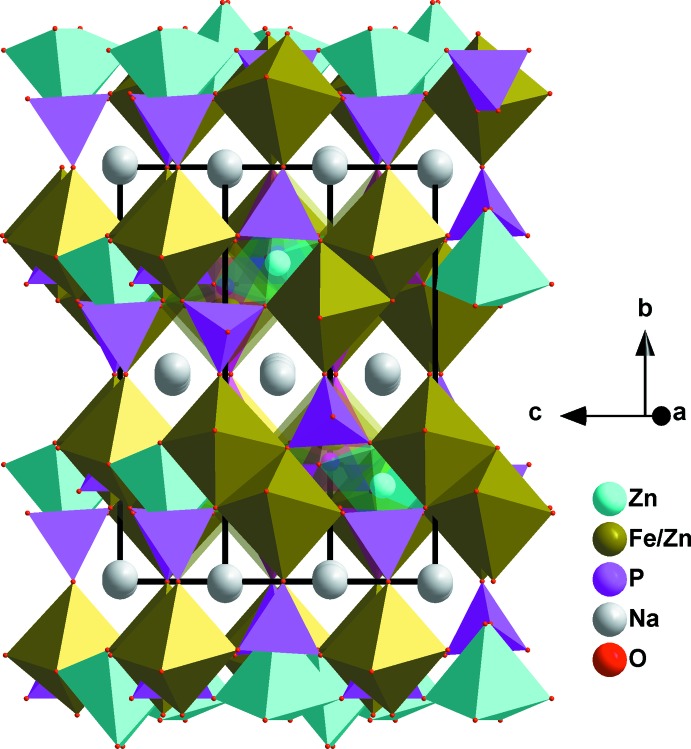
A stack of layers perpendicular to the *b* axis, showing small tunnels along the [201] direction.

**Figure 4:**
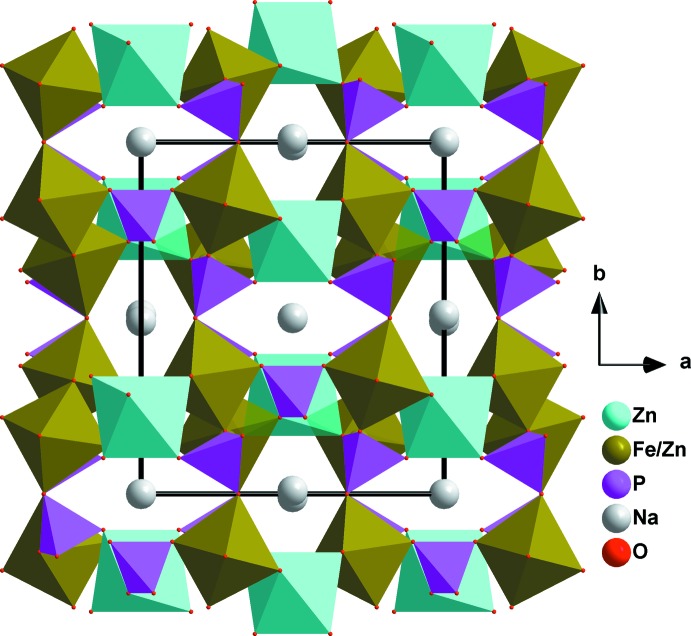
Polyhedral representation of Na_1.67_Zn_1.67_Fe_1.33_(PO_4_)_3_ showing tunnels running along the [001] direction.

The crystal structure of this phosphate compound consists of infinite kinked chains of two edge-sharing [Fe1/Zn1O_6_] octa­hedra leading to the formation of [(Fe1,Zn1)_2_O_10_] dimers that are connected by a common edge to [Zn2O_6_] octa­hedra, as shown in Fig. 2 ▸. These chains are linked by PO_4_ tetra­hedral groups, forming a stack of sheets perpendicular to [010] and alternating with sodium layers, as shown in Fig. 3 ▸, which reveal small tunnels along the [201] direction. The three-dimensional framework also encloses two types of large tunnels, in which the Na^+^ cations reside, as shown in Fig. 4 ▸. The site 4*e* centred on the first tunnel is partially occupied by Na1 [0.332 (3)], whereas Na2 occupies site 4*a* centred on the second tunnel. Each sodium atom is surrounded by eight oxygen atoms with Na1—O and Na2—O bond lengths in the ranges 2.448 (1)–2.908 (2) Å, and 2.324 (1)–2.901 (1) Å, respectively. The displacement ellipsoids of the partially occupied atom Na1 are rather larger than those of the rest of the atoms. Most probably this is due to the size of the channels, which allows atom Na1 to have more freedom. The disorder of Na in the tunnel may presage ionic mobility for this material.

## Synthesis and crystallization   

Single crystals of Na_1.67_Zn_1.67_Fe_1.33_(PO_4_)_3_ were synthesised by conventional solid-state reaction (Girolami *et al.*, 1999 ▸). The nitrate-based sodium, zinc and iron precursors, in addition to the 85 wt% H_3_PO_4_ were taken in proportions corresponding to the molar ratio Na:Zn:Fe:P = 2:2:1:3. The resulting reaction mixture was ground in an agate mortar and progressively heated in a platinum crucible to the melting temperature of 1135 K. The melted product was cooled at a rate of 5 K/h. The product was obtained as transparent brown crystals corresponding to the title phosphate.

## Refinement   

Crystal data, data collection and structure refinement details are summarized in Table 1 ▸. Refinements of the site-occupancy factors of the metal site 8*f* revealed the ratio of Fe1:Zn1 = 0.668 (3):0.332 (3), whereas the the occupancy fraction of Na1 was constrained to that of Zn1 in order to maintain electrical neutrality. The highest peak and the deepest hole in the final difference Fourier map are at 0.72 and 0.40 Å from O1 and Zn2, respectively.

## Supplementary Material

Crystal structure: contains datablock(s) I. DOI: 10.1107/S2056989015009767/pk2551sup1.cif

Structure factors: contains datablock(s) I. DOI: 10.1107/S2056989015009767/pk2551Isup2.hkl

CCDC reference: 1402019

Additional supporting information:  crystallographic information; 3D view; checkCIF report

## Figures and Tables

**Table 1:** Experimental details

Crystal data	
Chemical formula	Na_1.67_Zn_1.67_Fe_1.33_(PO_4_)_3_
*M*_r_	506.59
Crystal system, space group	Monoclinic, *C*2/*c*
Temperature (K)	296
*a*, *b*, *c* ()	11.7545(4), 12.5080(4), 6.4014(2)
()	113.507(1)
*V* (^3^)	863.06(5)
*Z*	4
Radiation type	Mo *K*
(mm^1^)	7.52
Crystal size (mm)	0.31 0.25 0.19

Data collection	
Diffractometer	Bruker X8 APEX
Absorption correction	Multi-scan (*SADABS*; Bruker, 2009 ▸)
*T*_min_, *T*_max_	0.504, 0.748
No. of measured, independent and observed [*I* > 2(*I*)] reflections	18880, 2101, 1997
*R*_int_	0.031
(sin /)_max_ (^1^)	0.833

Refinement	
*R*[*F* ^2^ > 2(*F* ^2^)], *wR*(*F* ^2^), *S*	0.017, 0.046, 1.19
No. of reflections	2101
No. of parameters	96
_max_, _min_ (e ^3^)	0.65, 1.21

Computer programs: *APEX2* and *SAINT* (Bruker, 2009 ▸), *SHELXS97* and *SHELXL97* (Sheldrick, 2008 ▸), *ORTEPIII* (Burnett Johnson, 1996 ▸), *ORTEP-3 for Windows* Farrugia, 2012 ▸), *DIAMOND* (Brandenburg, 2006 ▸) and *publCIF* (Westrip, 2010 ▸).

## supplementary crystallographic information

### Crystal data

**Table:**

Na_1.67_Zn_1.67_Fe_1.33_(PO_4_)_3_	*F*(000) = 977
*M**_r_* = 506.59	*D*_x_ = 3.904 Mg m^−^^3^
Monoclinic, *C*2/*c*	Mo *K*α radiation, λ = 0.71073 Å
Hall symbol: -c 2yc	Cell parameters from 2101 reflections
*a* = 11.7545 (4) Å	θ = 2.5–36.3°
*b* = 12.5080 (4) Å	µ = 7.52 mm^−^^1^
*c* = 6.4014 (2) Å	*T* = 296 K
β = 113.507 (1)°	Block, brown
*V* = 863.06 (5) Å^3^	0.31 × 0.25 × 0.19 mm
*Z* = 4	

### Data collection

**Table:**

Bruker X8 APEX diffractometer	2101 independent reflections
Radiation source: fine-focus sealed tube	1997 reflections with *I* > 2σ(*I*)
Graphite monochromator	*R*_int_ = 0.031
φ and ω scans	θ_max_ = 36.3°, θ_min_ = 2.5°
Absorption correction: multi-scan (*SADABS*; Bruker, 2009)	*h* = −19→16
*T*_min_ = 0.504, *T*_max_ = 0.748	*k* = −20→20
18880 measured reflections	*l* = −10→10

### Refinement

**Table:**

Refinement on *F*^2^	0 restraints
Least-squares matrix: full	*w* = 1/[σ^2^(*F*_o_^2^) + (0.016*P*)^2^ + 1.8532*P*] where *P* = (*F*_o_^2^ + 2*F*_c_^2^)/3
*R*[*F*^2^ > 2σ(*F*^2^)] = 0.017	(Δ/σ)_max_ = 0.001
*wR*(*F*^2^) = 0.046	Δρ_max_ = 0.65 e Å^−^^3^
*S* = 1.19	Δρ_min_ = −1.20 e Å^−^^3^
2101 reflections	Extinction correction: *SHELXL97* (Sheldrick, 2008), Fc^*^=kFc[1+0.001xFc^2^λ^3^/sin(2θ)]^-1/4^
96 parameters	Extinction coefficient: 0.0027 (2)

### Special details

**Table:**

Geometry. All e.s.d.'s (except the e.s.d. in the dihedral angle between two l.s. planes) are estimated using the full covariance matrix. The cell e.s.d.'s are taken into account individually in the estimation of e.s.d.'s in distances, angles and torsion angles; correlations between e.s.d.'s in cell parameters are only used when they are defined by crystal symmetry. An approximate (isotropic) treatment of cell e.s.d.'s is used for estimating e.s.d.'s involving l.s. planes.
Refinement. Reflections were merged by *SHELXL* according to the crystal class for the calculation of statistics and refinement._reflns_Friedel_fraction is defined as the number of unique Friedel pairs measured divided by the number that would be possible theoretically, ignoring centric projections and systematic absences.

### Fractional atomic coordinates and isotropic or equivalent isotropic displacement parameters (Å^2^)

**Table:**

	*x*	*y*	*z*	*U*_iso_*/*U*_eq_	Occ. (<1)
Fe1	0.71738 (2)	0.84648 (2)	0.12925 (3)	0.00552 (5)	0.668 (3)
Zn1	0.71738 (2)	0.84648 (2)	0.12925 (3)	0.00552 (5)	0.332 (3)
Zn2	0.5000	0.73133 (2)	0.2500	0.00966 (5)	
P1	0.76212 (3)	0.60983 (2)	0.37448 (5)	0.00388 (6)	
P2	0.5000	0.28835 (3)	0.2500	0.00330 (7)	
Na1	1.0000	0.49141 (19)	0.7500	0.0356 (7)	0.664 (6)
Na2	0.5000	0.5000	0.0000	0.01511 (18)	
O1	0.83510 (9)	0.66524 (7)	0.60760 (15)	0.00695 (15)	
O2	0.77771 (9)	0.67779 (8)	0.18481 (15)	0.00760 (15)	
O6	0.45837 (8)	0.21761 (8)	0.03327 (15)	0.00622 (15)	
O3	0.62448 (9)	0.60224 (8)	0.32496 (16)	0.00806 (16)	
O5	0.39712 (9)	0.36396 (8)	0.24820 (16)	0.00747 (16)	
O4	0.82109 (10)	0.49950 (8)	0.38406 (18)	0.01105 (17)	

### Atomic displacement parameters (Å^2^)

**Table:**

	*U*^11^	*U*^22^	*U*^33^	*U*^12^	*U*^13^	*U*^23^
Fe1	0.00523 (7)	0.00632 (7)	0.00583 (7)	−0.00082 (5)	0.00307 (5)	−0.00067 (5)
Zn1	0.00523 (7)	0.00632 (7)	0.00583 (7)	−0.00082 (5)	0.00307 (5)	−0.00067 (5)
Zn2	0.01124 (10)	0.00917 (10)	0.01083 (10)	0.000	0.00679 (8)	0.000
P1	0.00555 (12)	0.00355 (11)	0.00280 (11)	−0.00051 (9)	0.00192 (9)	−0.00023 (8)
P2	0.00319 (15)	0.00365 (15)	0.00256 (15)	0.000	0.00063 (12)	0.000
Na1	0.0167 (8)	0.0452 (13)	0.0345 (11)	0.000	−0.0008 (7)	0.000
Na2	0.0221 (4)	0.0079 (3)	0.0091 (3)	0.0024 (3)	−0.0004 (3)	0.0006 (3)
O1	0.0098 (4)	0.0072 (4)	0.0035 (3)	−0.0020 (3)	0.0022 (3)	−0.0015 (3)
O2	0.0090 (4)	0.0101 (4)	0.0043 (3)	−0.0022 (3)	0.0032 (3)	0.0013 (3)
O6	0.0057 (3)	0.0081 (4)	0.0044 (3)	−0.0004 (3)	0.0015 (3)	−0.0024 (3)
O3	0.0068 (4)	0.0083 (4)	0.0101 (4)	−0.0015 (3)	0.0044 (3)	−0.0006 (3)
O5	0.0055 (3)	0.0064 (3)	0.0092 (4)	0.0012 (3)	0.0016 (3)	−0.0029 (3)
O4	0.0147 (4)	0.0062 (4)	0.0125 (4)	0.0026 (3)	0.0057 (3)	−0.0019 (3)

### Geometric parameters (Å, º)

**Table:**

Fe1—O5^i^	1.9514 (10)	P2—O6	1.5510 (9)
Fe1—O4^ii^	1.9607 (10)	P2—O6^vi^	1.5510 (9)
Fe1—O1^iii^	2.0170 (10)	Na1—O4^ix^	2.4476 (11)
Fe1—O2^iv^	2.0567 (10)	Na1—O4	2.4476 (11)
Fe1—O6^v^	2.0684 (9)	Na1—O4^x^	2.5713 (12)
Fe1—O2	2.2091 (10)	Na1—O4^vii^	2.5713 (12)
Zn2—O3^vi^	2.1019 (10)	Na1—O1	2.812 (2)
Zn2—O3	2.1019 (10)	Na1—O1^ix^	2.812 (2)
Zn2—O6^vii^	2.1549 (10)	Na1—O6^xi^	2.908 (2)
Zn2—O6^v^	2.1549 (10)	Na1—O6^xii^	2.908 (2)
Zn2—O1^iii^	2.2028 (9)	Na2—O5^xiii^	2.3239 (9)
Zn2—O1^viii^	2.2028 (9)	Na2—O5^vi^	2.3239 (9)
P1—O3	1.5225 (10)	Na2—O3	2.3823 (9)
P1—O4	1.5345 (10)	Na2—O3^v^	2.3824 (9)
P1—O2	1.5518 (10)	Na2—O3^xiii^	2.5179 (10)
P1—O1	1.5563 (9)	Na2—O3^vi^	2.5179 (10)
P2—O5	1.5317 (10)	Na2—O5^v^	2.9008 (10)
P2—O5^vi^	1.5317 (10)	Na2—O5	2.9008 (10)
			
O5^i^—Fe1—O4^ii^	95.89 (4)	O4—Na1—O1	55.92 (4)
O5^i^—Fe1—O1^iii^	109.05 (4)	O4^x^—Na1—O1	114.04 (7)
O4^ii^—Fe1—O1^iii^	88.00 (4)	O4^vii^—Na1—O1	61.59 (4)
O5^i^—Fe1—O2^iv^	87.13 (4)	O4^ix^—Na1—O1^ix^	55.92 (4)
O4^ii^—Fe1—O2^iv^	101.39 (4)	O4—Na1—O1^ix^	119.76 (8)
O1^iii^—Fe1—O2^iv^	160.53 (4)	O4^x^—Na1—O1^ix^	61.58 (4)
O5^i^—Fe1—O6^v^	161.52 (4)	O4^vii^—Na1—O1^ix^	114.04 (7)
O4^ii^—Fe1—O6^v^	100.93 (4)	O1—Na1—O1^ix^	78.70 (7)
O1^iii^—Fe1—O6^v^	79.35 (4)	O4^ix^—Na1—O6^xi^	114.24 (8)
O2^iv^—Fe1—O6^v^	82.12 (4)	O4—Na1—O6^xi^	70.35 (5)
O5^i^—Fe1—O2	79.41 (4)	O4^x^—Na1—O6^xi^	83.40 (5)
O4^ii^—Fe1—O2	173.23 (4)	O4^vii^—Na1—O6^xi^	101.22 (6)
O1^iii^—Fe1—O2	88.95 (4)	O1—Na1—O6^xi^	125.27 (3)
O2^iv^—Fe1—O2	83.34 (4)	O1^ix^—Na1—O6^xi^	144.38 (3)
O6^v^—Fe1—O2	84.43 (4)	O4^ix^—Na1—O6^xii^	70.35 (5)
O3^vi^—Zn2—O3	79.62 (5)	O4—Na1—O6^xii^	114.24 (8)
O3^vi^—Zn2—O6^vii^	92.79 (4)	O4^x^—Na1—O6^xii^	101.22 (6)
O3—Zn2—O6^vii^	113.99 (4)	O4^vii^—Na1—O6^xii^	83.40 (5)
O3^vi^—Zn2—O6^v^	113.99 (4)	O1—Na1—O6^xii^	144.38 (3)
O3—Zn2—O6^v^	92.79 (4)	O1^ix^—Na1—O6^xii^	125.27 (3)
O6^vii^—Zn2—O6^v^	145.51 (5)	O6^xi^—Na1—O6^xii^	51.96 (5)
O3^vi^—Zn2—O1^iii^	164.40 (4)	O5^xiii^—Na2—O5^vi^	180.00 (3)
O3—Zn2—O1^iii^	86.51 (4)	O5^xiii^—Na2—O3	100.43 (3)
O6^vii^—Zn2—O1^iii^	86.30 (4)	O5^vi^—Na2—O3	79.57 (3)
O6^v^—Zn2—O1^iii^	73.53 (3)	O5^xiii^—Na2—O3^v^	79.57 (3)
O3^vi^—Zn2—O1^viii^	86.51 (4)	O5^vi^—Na2—O3^v^	100.43 (3)
O3—Zn2—O1^viii^	164.40 (4)	O3—Na2—O3^v^	180.0
O6^vii^—Zn2—O1^viii^	73.53 (3)	O5^xiii^—Na2—O3^xiii^	107.29 (3)
O6^v^—Zn2—O1^viii^	86.30 (4)	O5^vi^—Na2—O3^xiii^	72.71 (3)
O1^iii^—Zn2—O1^viii^	108.06 (5)	O3—Na2—O3^xiii^	113.43 (4)
O3—P1—O4	112.20 (6)	O3^v^—Na2—O3^xiii^	66.57 (4)
O3—P1—O2	108.58 (5)	O5^xiii^—Na2—O3^vi^	72.71 (3)
O4—P1—O2	109.36 (6)	O5^vi^—Na2—O3^vi^	107.29 (3)
O3—P1—O1	111.16 (6)	O3—Na2—O3^vi^	66.57 (4)
O4—P1—O1	107.14 (6)	O3^v^—Na2—O3^vi^	113.43 (4)
O2—P1—O1	108.32 (5)	O3^xiii^—Na2—O3^vi^	180.00 (3)
O5—P2—O5^vi^	103.73 (8)	O5^xiii^—Na2—O5^v^	53.55 (4)
O5—P2—O6	112.27 (5)	O5^vi^—Na2—O5^v^	126.45 (4)
O5^vi^—P2—O6	109.00 (5)	O3—Na2—O5^v^	85.32 (3)
O5—P2—O6^vi^	109.00 (5)	O3^v^—Na2—O5^v^	94.68 (3)
O5^vi^—P2—O6^vi^	112.28 (5)	O3^xiii^—Na2—O5^v^	67.11 (3)
O6—P2—O6^vi^	110.43 (7)	O3^vi^—Na2—O5^v^	112.89 (3)
O4^ix^—Na1—O4	175.26 (12)	O5^xiii^—Na2—O5	126.45 (4)
O4^ix^—Na1—O4^x^	79.21 (3)	O5^vi^—Na2—O5	53.55 (4)
O4—Na1—O4^x^	100.58 (3)	O3—Na2—O5	94.68 (3)
O4^ix^—Na1—O4^vii^	100.58 (3)	O3^v^—Na2—O5	85.32 (3)
O4—Na1—O4^vii^	79.20 (3)	O3^xiii^—Na2—O5	112.89 (3)
O4^x^—Na1—O4^vii^	174.93 (11)	O3^vi^—Na2—O5	67.11 (3)
O4^ix^—Na1—O1	119.76 (8)	O5^v^—Na2—O5	180.00 (3)

Symmetry codes: (i) *x*+1/2, *y*+1/2, *z*; (ii) −*x*+3/2, *y*+1/2, −*z*+1/2; (iii) −*x*+3/2, −*y*+3/2, −*z*+1; (iv) −*x*+3/2, −*y*+3/2, −*z*; (v) −*x*+1, −*y*+1, −*z*; (vi) −*x*+1, *y*, −*z*+1/2; (vii) *x*, −*y*+1, *z*+1/2; (viii) *x*−1/2, −*y*+3/2, *z*−1/2; (ix) −*x*+2, *y*, −*z*+3/2; (x) −*x*+2, −*y*+1, −*z*+1; (xi) *x*+1/2, −*y*+1/2, *z*+1/2; (xii) −*x*+3/2, −*y*+1/2, −*z*+1; (xiii) *x*, −*y*+1, *z*−1/2.
